# Measurement properties of the Spanish version of the brief resilient coping scale (BRCS) in cancer patients

**DOI:** 10.1016/j.ijchp.2022.100313

**Published:** 2022-05-31

**Authors:** Caterina Calderon, Urbano Lorenzo-Seva, Pere J. Ferrando, Elena Sorribes, Adán Rodríguez-González, Berta M. Obispo, Luka Mihic-Góngora, María J. Corral, Jacobo Rogado, Patricia Cruz-Castellanos, Paula Jiménez-Fonseca

**Affiliations:** a Department of Clinical Psychology and Psychobiology, Faculty of Psychology, University of Barcelona, Spain; b Department of Psychology, Faculty of Psychology, Rovira and Virgili University, Tarragona, Spain; c Department of Social Psychology and Quantitative, Faculty of Psychology, University of Barcelona, Spain; d Department of Medical Oncology, Hospital Universitario Infanta Leonor, Madrid, Spain; e Department of Medical Oncology, Hospital Universitario La Paz, Madrid, Spain; f Department of Medical Oncology, Hospital Universitario Central of Asturias, Oviedo, Spain

**Keywords:** Invariance, Factor analysis, Resilience, Oncology, Multigroup analysis, Instrumental study

## Abstract

**Background/Objective:**

Resilience is the capacity to adaptively confront stress. The aim of this study was to evaluate the psychometric properties, convergent validity, and factorial invariance of the Spanish version of the Brief Resilient Coping Scale (BRCS).

**Method:**

Exploratory and confirmatory factor analyses based on a cross-validation were conducted to explore the scale's dimensionality and test for strong (scalar) measurement invariance across gender, age, tumor site, and survival, by fitting multiple-group confirmatory solutions. An extended structural equation model was used to assess external validity. Prospective, multicenter cohort study of 636 patients who completed the BRCS, Satisfaction with Life Scale (SWLS), and Spiritual well‐being (FACIT-sp) scales.

**Results:**

The data supported a unidimensional structure. The BRCS is a very short, narrow bandwidth measure, with items demonstrating high discriminating power. A strong invariance solution demonstrated excellent fit across gender, age, tumor site, and survival. Scores derived from the unidimensional structure exhibited satisfactory degrees of reliability (ω = .86) and determinacy (FDI = .94). BRCS revealed substantial associations with satisfaction with life and spirituality well-being (all *p* < .001), factors widely related to resilience, particularly in cancer patients.

**Conclusions:**

The Spanish version of the BRCS is a reliable, valid resilience measure in advanced cancer.

Psychological resilience has been defined as the capacity to adapt to stressful factors in a positive way, bounce back from adversity and find meaning in traumatic experience (Lau Ming et al., 2021). Originally, resilience was used in the field of physics to refer to the ability a material has to return to a state of equilibrium after being displaced and has been adapted in psychology as a theoretical construct of the process of protection, promotion, and recovery of mental health (Lau Khoo et al., 2021; Lau Ming et al., 2021). It has also been associated with a series of internal (for instance satisfaction, optimism, acceptance) and external (for example, perceived social support or coping strategies) psychological factors, as well as a variety of health and behavioral-related factors, such as lifestyle, exercise, perception of disease, and treatment compliance (Calderon et al., 2021; Lau Khoo et al., 2021; Oliveira et al., 2021).

Empirical evidence suggests that highly resilient cancer patients function better, have a higher pain threshold (Liesto et al., 2020), suffer less anxiety and depression (Çakir et al., 2021; Lau Ming et al., 2021), and adapt better to their social surroundings (Alarcón et al., 2020), and enjoy greater spiritual wellbeing (Çakir et al., 2021; Schwalm et al., 2021). Oncology and other immunosuppressed patients are at higher risk for severe complications due to the virus compared to healthy individuals (Curigliano et al., 2020; Ramanathan et al., 2020). In this context, resilience is vital to help patients confront the challenges of the disease, such as the negative emotions surrounding a cancer diagnosis, the perception of risk, and treatment compliance.

Evaluating resilience and resilience-associated variables, requires standardized measures with suitable psychometric properties. Several transcultural studies have found that the Brief Resilient Coping Scale (BRCS) exhibits good psychometric properties (Chmitorz et al., 2018; Fung, 2020). Resilience has largely been quantified with self-report measures, such as the Brief Resilient Coping Scale (BRCS; Sinclair & Wallston, 2004). The BRCS is a 4-item, supposedly unidimensional scale designed to assess resources for adaptive coping with stress (Sinclair & Wallston, 2004). Scores display an internal consistency of the order of (*α* = .76) and test-retest reliability (*r* = .71) in specific samples, like medical (Heinen et al., 2017) nursing (Edo-Gual et al., 2015) students, individuals with systemic lupus (López-Pina et al., 2016), US service people (Rice & Liu, 2016), or the elderly (Cosco et al., 2016). The first adaptation to Spanish of the BCRS in a sample of 133 older people and yielded adequate validity and reliability (Tomás et al., 2012). Similarly, it has proven to be suitable for quantifying resilience in patients with chronic disease and exhibit psychometric robustness for continued use in older populations (Cosco et al., 2016), as well as in the German (Kocalevent et al., 2017) and Italian (Murphy et al., 2021) general population.

Despite having shown evidence for the use of the BCRS in different populations (Cosco et al., 2016; Rice & Liu, 2016; Tomás et al., 2012), few studies have probed the invariance of the measurement as a function of age and gender. Measurement invariance ensures that rating tools actually measure the same construct and with the same properties in sub-populations derived from the target population to which the instrument is addressed, for instance by gender and age (Putnick & Bornstein, 2016). In earlier studies, the BCRS has proven to be invariant to gender or age (Kocalevent et al., 2017), although there are studies that have found that resilience gradually increases with age (Murphy et al., 2021), except for the senior age bracket (Kocalevent et al., 2017), while others find no significant difference by age group (López-Pina et al., 2016). As for gender, men score higher on resilience than women (Kocalevent et al., 2017).

To the best of our knowledge, no psychometric studies of the BDRS score structure and properties have been conducted in Spanish patients with advanced cancer and we believe that this type of study is of clear interest given the importance of promoting resilience as a valuable resource in the psychological treatment of cancer. In principle, the properties of unidimensionality and measurement accuracy attained in previous studies can vary depending on the target population (e.g., Hambleton & Swaminathan, 1985) and can therefore not simply be extrapolated to our population of interest.

On the basis of these considerations, the initial aims of the instrumental study (Carretero-Dios & Pérez, 2007) are (i) to appraise the Spanish version of the BCRS factorial structure in patients with advanced cancer, (ii) assess the measurement invariance of BCRS scores in groups derived from the target population and defined by gender, age, tumor site, and survival, (iii) probe the suitability and accuracy of the measure (both marginal and conditional) in this kind of population, and (iv) appraise the external validity of the BCRS satisfaction with life and spiritual well‐being scores.

## Method

### Participants

A total of 636 patients participated in this study, of whom 338 (53.1%) were male, 298 (46.9%) female. Mean age was 64.9 years (*SD* = 10.3). Most were married or partnered (76.8%) and had a primary level of education (49.2%). All were retired or unemployed. As for clinical characteristics, the most common tumors were bronchopulmonary (31.8%), colorectal (15.6%), pancreatic (9.1%), breast (6.4%), and stomach (5.7%). Adenocarcinoma histology was the most prevalent (62.1%) and most cancers were stage IV (80.7%). The most common treatment was chemotherapy (53.1%), chemotherapy with a targeted drug (14.2%) and chemotherapy with immunotherapy (12.1%), immunotherapy (7.2%), and targeted drug (6.9%). Estimated survival was less than 18 months in 46.5% of the sample (see Table 1).

**Table 1:** Sociodemographic and clinical characteristics (*N*= 636).

Characteristics	*n* (%)
Sex	
Male	338 (53.1)
Female	298 (46.9)
Age	
≤55 years	128 (20.1)
56-65	223 (35.1)
≥ 66	285 (44.8)
Marital Status	
Married/ partnered	489 (76.8)
Not partnered	147 (23.1)
*p* value	
Educational level	
Primary	313 (49.2)
High school or higher	323 (50.8)
Cancer type	
Thoracic	206 (32.4)
Digestive	247 (38.8)
Others	183 (28.8)
Histology	
Adenocarcinoma	395 (62.1)
Others	241 (37.9)
Cancer stage	
Locally advanced	123 (19.3)
Metastatic disease (IV)	513 (80.7)
Estimated survival	
<18 months	296 (46.5)
≥18 months	340 (53.5)
Type of treatment	
Chemotherapy	338 (53.1)
Chemo+Targeted	90 (14.2)
Chemo+Immunotherapy	77 (12.1)
Immunotherapy	46 (7.2)
Targeted	44 (6.9)
Others	2 (0.3)

Mundfrom et al. (2005) performed a simulation study that helped define appropriate sample sizes for factor analysis. Given that our test has four items and a single factor, even in the worst case scenario of having a low communality, our analysis should be made with at least 95 observations. We sought to have a much larger sample to be able to divide the sample into different groups and still to have a reasonable sample size in each subsample for factor analysis. The subsample that was found to be the smallest was “Cancer type-others”. This subsample comprised 183 participants, still well above the size recommended by Mundfrom et al.

To further verify that the collected sample size was sufficient for the purposes of the research, we also undertook a power analysis for all the solutions fitted in the study by using the approach proposed by Lee et al. (2012). In all cases, the power to detect small to moderate misspecifications exceeded .90.

### Measures

Participants’ socio-demographic characteristics were collected using a standardized, self-report form. Information pertinent to participants’ disease was gleaned from medical records.

The Brief Resilient Coping Scale (BRCS) is a 4-item instrument (Sinclair & Wallston, 2004); we used the Spanish version of the BRCS (Tomás et al., 2012) for this study. The BRCS supposedly unidimensional outcome measure designed to capture to what extent an individual cope with stress and rebounds from it. Items have five possible responses; 1 means the statement “does not describe you at all” and 5 means “it describes you very well” (Appendix A). The sum score ranges from 4 to 20; the higher the score, the more resilience. The estimated internal consistency coefficient for the original scale was .69 (Sinclair & Wallston, 2004).

Satisfaction with Life Scale (SWLS) consists of 5 items and was developed to quantify the judgmental component of subjective well-being (Diener et al., 1985). Participants indicate how much they agree or disagree with each item on a 7-point scale ranging from 7 (*strongly agree*) to 1 (*strongly disagree*). The internal consistency of the SWLS scores in a sample of Spanish cancer patients was (α = .91) (Lorenzo-Seva et al., 2019).

Spiritual well‐being was gauged with the validated Spanish version of the Functional Assessment of Chronic Illness Therapy-Spiritual Well‐Being Scale (FACIT‐Sp; Jimenez-Fonseca et al., 2018; Peterman et al., 2002), a 12-item instrument scored on a five‐point scale and containing two subscales Meaning/Peace and Faith. The sum yields the index of spiritual well-being; the higher the score, the greater the person's wellbeing. Reliability ranged from .85-.86 in the Spanish sample (Jimenez-Fonseca et al., 2018).

### Procedure

A multi-institutional, prospective, observational study was conducted with the participation of 15 hospitals in Spain from February 2020 to November 2021. The study is part of a cancer patient research program funded by the Bioethics Group of the Spanish Society of Medical Oncology (SEOM). The study was approved by the Ethics Committee of each institution and by the Spanish Agency of Medicines and Medical Devices (AEMPS; identification code: ES14042015). Participants were 18 years of age or older with resected, histologically confirmed advanced cancer who were ineligible for surgery or other therapy. Individuals with any serious mental illness that kept them from comprehending the survey were excluded. Of the 663 individuals recruited, 636 were eligible. A total of 27 were excluded (6 failed to meet the inclusion criteria; 5 met an exclusion criterion, and 16 had incomplete data). Those who agreed to participate signed the informed consent form, were given instructions on how to fill in the written questionnaires, completed it at home, and handed them in to the auxiliary staff at the next visit. Data collection procedures were similar at all hospitals and data relating to the participants were obtained from the treating institutions. Participation was voluntary, anonymous, and did not affect patient care. Data were collected and updated by the medical oncologist, through a web-based platform (www.neoetic.es).

### Data analyses

Analyses were performed sequentially in keeping with the purposes stated above (Ferrando et al., 2022; Hernández-dorado et al., 2021). The total sample was split in half using Solomon procedure (Lorenzo-Seva, 2021) The first subsample was analyzed with an exploratory factor analysis (EFA) using FACTOR software (Ferrando & Lorenzo-seva, 2017). The second subsample was analyzed with a Confirmatory Factor Analysis (CFA) using MPLUS. Overall, and given the conditions of the study, i.e., few items, few response categories, relatively large sample, and some extreme item distributions, the item scores were treated as ordered-categorical variables; therefore, all the structural analyses at the item level (exploratory, confirmatory, multiple-group, and validity extended) were (a) based on the polychoric inter-item correlations and (b) fitted using robust weighted least squares estimation as implemented in FACTOR] and Mplus programs. Model fit and appropriateness were evaluated using three groups of indices that assessed different facets of fit: absolute fit (GFI and RMSR), relative fit (RMSEA), and comparative fit (CFI). As for reference values, GFI and CFI values ≥ .95 are indicative of good model fit (Schermelleh-Engel et al., 2003), whereas SRMR values ≤0.08 and RMSEA values ≤ .06 are deemed indicative of satisfactory fit. Descriptive analyses were conducted for BRCS and explored means standard deviations and distributions of the item score.

To assess the adequacy of matrix correlation to be factor analyzed, Kaiser-Meyer-Olkin (KMO) test for sampling adequacy was computed. Normed-MSA indices were also examined to determine if any item was not sharing enough communality with the entire set of items: Normed-MSA values below 0.50 suggest that the item does not measure the same domain as the remaining items in the pool and, hence, should be removed (Lorenzo-Seva & Ferrando, 2021). Optimal Implementation of Parallel Analysis was computed in order to assess the advised number of factors to be extracted: the percentage of explained common variance in the polychoric correlation matrix was estimated based on Minimum Rank Factor Analysis, and the noise solutions were obtained using independent column permutation of observed participants’ scores. The following indices were proved to appraise essential unidimensionality: Unidimensional Congruence (UniCo), Explained Common Variance (ECV), and Mean of item residual absolute loadings (MIREAL). UNICO values exceeding .95, ECV > .85, and MIREAL < .30 suggest that the data can be treated as essentially unidimensional. Next, to study the replicability of the factor structure obtained in the first Solomon subsample, a CFA was carried out on the second subsample using the procedure and criteria described thus far. Finally, as both previous analyses led to the same conclusions, the common CFA solution was fitted to the total sample to use all the information available from the data.

The common restricted CFA solution was obtained consistently in all the analyses and was used to assess invariance in groups defined by: gender (two groups), age (three groups), tumor site (three groups), and survival (two groups). The aim was to achieve strong (scalar) measurement invariance in all the comparisons. When this condition is attained, it can be then assumed that the BCRS dimensionality and structure is the same in the different groups; consequently, observed mean differences can validly be interpreted as reflecting ‘true’ mean group differences. All the multiple-group solutions were fitted using the general procedure described. In all cases, we fitted weaker-invariance solutions (non-invariant loadings, thresholds, or both) prior to fitting the strong target solutions, and tested the appropriateness of the assumed simpler strong-invariance solutions.

The BRCS is a very short, narrow-bandwidth measure, the items of which would possibly attain high discriminating power (see Reise et al., 2021). What we want to see here is (a) whether this property can compensate for the scant number of items, (b) if the usual sum scores are accurate indicators of the construct they measure, and (c) the range of trait levels at which the BRCS scores provide accurate measurement in the target population. This questions were answered by using the factor determinacy index (FDI), marginal reliability estimate, and McDonald's omega reliability estimate (for point (a); the extent to which the items approached parallelism and the ordinal fidelity coefficient (see Ferrando & Lorenzo-Seva, 2021) for point (b), and the test information curve for point (c).

Finally, evidence of convergent validity based on the relations with theoretically relevant external variables was explored by fitting a structural equation model in which the CFA solution was extended to include the SWLS and FACIT scores to the data as external variables.

## Results

### Descriptive statistics and structure assessment

Descriptive statistics of the BRCS can be found in Table 2. Item scores ranged from 3.41 to 3.85. BRCS item score distributions were unimodal and asymmetrical, thereby indicating that most of the values were concentrated at the highest end of the response scale. All the corrected item-total correlations surpassed .61.

**Table 2:** Brief Resilient Coping Scale (BRCS) characteristics.

Items	*M*	*SD*	Skews.
1. I look for creative ways to alter difficult situations.	3.48	1.3	-0.41
2. Regardless of what happens to me, I believe I can control my reaction to it.	3.56	1.2	-0.38
3. I believe I can grow in positive ways by dealing with difficult situations.	3.85	1.1	-0.68
4. I actively look for ways to replace the losses I encounter in life.	3.41	1.3	-0.40
BRCS total	14.3	3.9	

### Exploratory factor analysis of the first Solomon subsample

The inter-item polychoric correlation matrix had good properties KMO = .80. Normed-MSA for items ranged from .75 to .85. These outcomes indicated that the correlation matrix is well suited for factorial analysis and that all four items contribute effectively to the common variance. Parallel analyses revealed that a single dimension accounted for 83.26% of the common variance and suggested a single factor to be extracted. In addition, the essential unidimensionality index values were UNICO = .990, ECV = .898, and MIREAL = .226. These outcomes also point toward a single dimension possibly being suitable for the dataset.

The unidimensional factor analysis solution yielded acceptable goodness-of-fit levels: RMSEA = .050, CFI = .998, GFI = .998, and RMSR = .026. The bootstrap 90% confidence intervals of loading values overlapped for three items (1, 2, and 4), while the corresponding confidence interval for item 3 (the one with the largest loading value) suggested that this item had a significantly larger loading value than the other three. Overall, the conclusion of the EFA was that the one-factor solution was the most acceptable for the BRCS items. Furthermore, the results indicated that three of the four items were parallel.

### Confirmatory factor analysis of the second Solomon subsample

Using Mplus, a restricted unidimensional solution was fitted in the second subsample, constraining items 1, 2, and 4 to have the same loading values. The fit was quite reasonable: GFI and RMSR estimates were .994 and .042, respectively, while RMSEA was .070 and CFI was .990.

### Factor analysis and psychometric properties of the total sample

Finally, inasmuch as the EFA and CFA of both subsamples lead to the same conclusion, the overall sample was re-analyzed using the restricted CFA solution above. The fit of this solution at the total sample level was, again, quite acceptable: GFI = .993, RMSR = .033, RMSEA = .048, and CFI = .993. As for loading estimates, the common loading for the three parallel items (1, 2, and 4) was 0.763 and the loading estimate for item 3 was .895. As expected, these values are quite high for a personality measure (especially in the case of item 3) and prognosticate that high measurement accuracy would be possible even when only four items are available.

### Measurement invariance and group differences

In all multiple group analyses, the strong invariance solution displayed an excellent fit. Preliminary, weaker-invariance solutions (non-invariant loadings, thresholds, or both) were tried and we found that the strong solution clearly fitted much better than less restricted solutions, both in relative terms (as indicated by the RMSEA), as well as according to the parsimony Bayesian information criterion index. Therefore, only, the overall fit results and group mean estimates are reported in Table 3.

**Table 3:** Test for invariance across gender, age group, tumor site, and survival.

Groups	*M*		*SD*		(90% *CI*)	χ^2^ (*df*)	CFI	RMSEA	(90% CI)
Gender						26.46 (21)	.998	.029	(.000, .058)
Men (fixed)	.00 (fixed)		1.00						
Women	-141		1.00		(-.288, .001)				
Age group (years)						49.08 (38)	.996	.037	(.000, .064)
Group 1 (≤ 60) (fixed)	.00 (fixed)		1.00						
Group 2 (60-70)	.06		1.00		(-.045, .165)				
Group 3 (≥ 70)	-.362		1.00		(-.477, -.247)				
Tumor site						34.53 (38)	.999	.001	(.000, .042)
Group 1 (thoracic)	.00 (fixed)		1.00						
Group 2 (digestive)	.011		1.00		(-.095, .117)				
Group 3 (others)	.026		1.00		(-.086, .138)				
Survival						31.18 (21)	.996	.039	(.000, .066)
Group 1 (< 18 month)	.00 (fixed)		1.00						
Group 2 (≥ 18 month)	0.252		1.00		(.101, .399)				

To interpret the mean differences in the table, we stress that the means are always fixed to zero in the first group for identification purposes and are freely estimated in the rest. There were significant age and survival effects associated with lower BRCS scores, particularly in patients ≥70 years old and those with estimated survival <18 months. No significant differences were found based on gender or tumor site.

### BCRS score properties and accuracy

Two types of scores were obtained for the BCRS based on the structural solution summarized above: EAP factor score estimates (Ferrando & Lorenzo-Seva, 2016) and the usual simple sum scores. For the first type of scores, FDI and marginal reliability were .94 and .89, respectively. As for the sum scores, the omega reliability estimate was 0.86. In both cases, the reliability estimate is quite high for such a short test. The estimated fidelity coefficient was .94, denoting that the usual sum scores are already good proxies for measuring the Resilience dimension.

**Figure 1:**
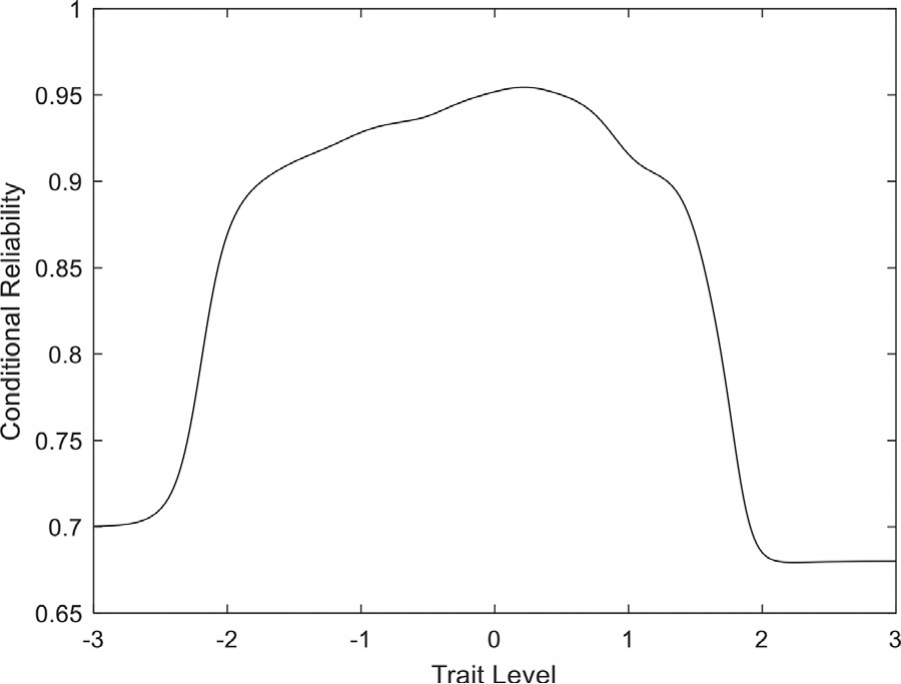
The reliability (conditional) estimates as a function of trait level.

Figure 1 shows the (conditional) reliability estimates as a function of the trait level; essentially, providing the BCRS information curve. The interpretation is quite clear: the BCRS scores are highly accurate along the trait range that contains most of the target population (between -2 and +2 in standardized values). This is a very positive feature of the scores, forasmuch as it implies that the scale measures resilience equally well at all levels.

### External validity assessment of sources

**Figure 2:**
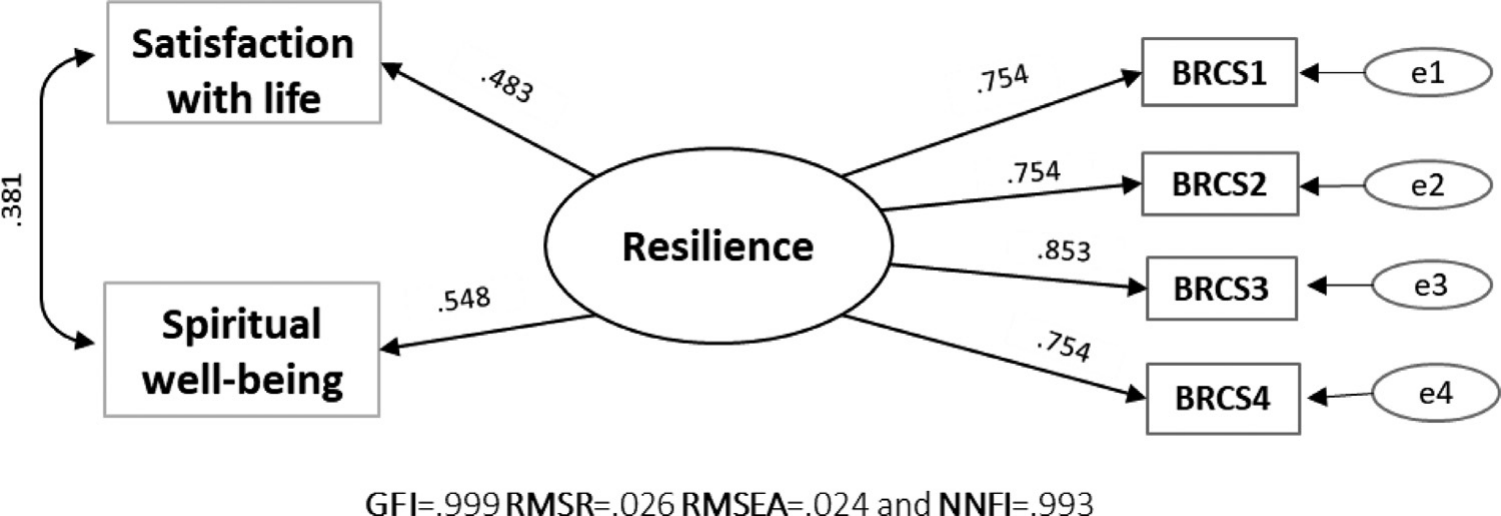
Path model and standardized factor weights of the 4 BRCS items in the sub-sample (*n* = 318). *Note:* Standardized coefficients are presented and all paths are significant at the .001 level.

The extended structural model based on the core invariant factorial solution obtained in stages 2 and 3 fit the data quite well: Goodness of Fit Index (GFI) = .999, Root Mean Square of Residuals (RMSR) = .026, Root Mean Square Error of Approximation (RMSEA) = .024, and Non-Normed Fit Index (NNFI) = .993, although slightly different from stage 2 because it is a sub-sample. Figure 2 provides the estimates. The BRCS associated substantially with satisfaction with life (*r* = .360) and spirituality well-being (*r* = .468), which are related closely with resilience, particularly in cancer patients.

## Discussion

This study of more than 630 individuals with advanced cancer endorses the BCRS as being reliable and valid to evaluate resilience in this population. The results of the EFA and CFA clearly substantiate that the BCRS is unidimensional: a dominant common factor fits well and explains 83% of the item common variance. Item loadings are high and, hence, the scale is well identified in spite of its brevity.

With respect to measurement invariance, scalar invariance is confirmed in all the groups analyzed: gender, age, tumor site, and survival, which allows both observed score means and latent estimated means to be validly compared (Gregorich, 2006). Males and females did not significantly vary as regards resilience, similar to an earlier study in patients with lupus erythematosus (López-Pina et al., 2016), but not in a study of the German general population that found men to be slightly more resilient than women (Kocalevent et al., 2017). However, significant differences were observed across age brackets. Generally, resilience tended to increase gradually with age, although there was an appreciable decline starting at 70 years of age, in line with Kovalant's study (Kocalevent et al., 2017). This decrease in resilience beginning at 70 years old might be explained by the greater number of health issues, diminished functionality, and greater anxiety and loneliness experienced by seniors (Rentscher et al., 2021; Schwalm et al., 2021). For researchers, this information is pertinent to identify those variables that can bolster resilience in the ever-aging Western population (Keating, 2022). While no significant differences were detected based on tumor site, there were disparities with respect to survival in the sense that those individuals whose survival expectancy is greatest are the most resilient. Empirical evidence suggests that, in cancer patients, higher levels of resilience correlate with greater hope and less anxiety and depression (Gao et al., 2019; Seiler & Jenewein, 2019).

Despite its brevity, the BCRS was seen to have good reliability, even for the simpler sum scores (ω = .86), with a good fidelity index (*FDI* = .94), evidences that the summary score is a good indicator of resilience. Furthermore, the scores provide accurate measurements across all resilience levels. Three of the items can be considered parallel, making the simple sum scores (unit weight) a suitable approach (Thissen & Greenberg, 1983) and endorse its applicability in different contexts and intercultural comparison (Kocalevent et al., 2017; López-Pina et al., 2016). Future studies should examine the BRCS psychometric properties in other clinical simples with chronic or degenerative diseases.

The association of the BCRS with spirituality and satisfaction with life is similar to findings of other studies that point toward the scale's good construct validity (Kocalevent et al., 2017; López-Pina et al., 2016). Resilience has been linked to less of a negative psychosocial impact and less existential angst brought about by advanced disease, death, and dying (Joyce et al., 2018; Lau Ming et al., 2021; López-Pina et al., 2016). Resilience is a response to adverse life events, such as the diagnosis of incurable cancer, and a means by which patients adapt as best they can to these circumstances (Seiler & Jenewein, 2019). Although individuals with incurable cancer may suffer high levels of anguish and personal disruption, studies indicate that those with greater resilience manage to give meaning to the experience that promotes a greater feeling of peace and acceptance (Schwalm et al., 2021; Seiler & Jenewein, 2019). Our findings suggest that the time is right for studies on advanced cancer and palliative care to undertake a holistic approach to work on factors such as tenacity, satisfaction, or positive growth nurtures resilient coping.

This study has a series of strengths and limitations. Its greatest strength is the analysis of the psychometric properties of the BRCS scale in a large sample of individuals with varying kinds of cancer, that has enabled us to divide the sample randomly into two for exploratory and confirmatory analyses. A potential limitation is its cross-sectional design, which does not allow for interpretations of causality or possible mediating effects. Further longitudinal evaluations of the BRCS are necessary to demonstrate its performance also in different clinical target populations. Finally, we must be cautious when interpreting these results, bearing in mind that all the subjects eligible to participate did so voluntarily, which may have introduced a self-selection bias.

To conclude, the Spanish version of the BRCS is a reliable and valid resilience measure in advanced cancer patients. In terms of applicability, the brevity of this 4-item scale makes it convenient in clinical contexts to avoid respondent fatigue when several other variables or constructs are to be measured. Given that resilience can be contemplated as a protective factor that helps individuals cope with a cancer diagnosis (Çakir et al., 2021; Lau, Ming et al., 2021). Intervention programs aimed at women with cancer should include strategies to develop resilience so as to enhance their mental health and quality of life (Joyce et al., 2018; Lau, Ming et al., 2021).

## Funding

This study was funded by the FSEOM (Spanish Society of Medical Oncology Foundation) grant for Projects of the Collaborative Groups in 2018 and by an Astra Zeneca grant (ES2020-1939). The sponsor of this research has not participated in data collection, analysis, or interpretation; in writing the report, or in the decision to submit the article for publication.

## Appendix A Brief Resilient Coping Scale (BRCS)

*Instructions: Consider how well the following statements describe your behavior and actions.*

1. I look for creative ways to alter difficult situations
2. Regardless of what happens to me, I believe I can control my reaction to it
3. I believe I can grow in positive ways by dealing with difficult situations
4. I actively look for ways to replace the losses I encounter in life

The format of a five-point Likert scale from 1 (*Does not describe me at all*) to 5 (*Describes me very well*).
